# Inhibition of ATM, ATR and DNA-PK radiosensitises 3D uveal melanoma models to X-rays and proton beam therapy

**DOI:** 10.3389/fonc.2026.1921423

**Published:** 2026-08-14

**Authors:** Laura Hawkins, Ryan Morris, Helen Kalirai, Karen Aughton, Rumana Hussain, Sarah E. Coupland, Jason L. Parsons

**Affiliations:** 1 Department of Cancer and Genomic Sciences, University of Birmingham, Edgbaston, Birmingham, United Kingdom; 2 Department of Eye and Vision Science, Institute of Life Course and Medical Science, University of Liverpool, Liverpool, United Kingdom; 3 Liverpool Ocular Oncology Centre, Liverpool University Hospitals Trust, Liverpool, United Kingdom; 4 School of Physics and Astronomy, University of Birmingham, Edgbaston, Birmingham, United Kingdom

**Keywords:** DNA damage repair, proton beam therapy, radiosensitisation, radiotherapy, uveal melanoma

## Abstract

**Introduction:**

Proton beam therapy (PBT) is a well-established treatment modality for uveal melanoma (UM), where it exerts its therapeutic effect primarily through the induction of significant DNA damage, particularly DNA double strand breaks (DSBs) that are the most challenging for the cell to repair. Following DSB induction, a coordinated repair response is orchestrated by the key protein kinases, ataxia telangiectasia mutated (ATM), ataxia telangiectasia and Rad3-related (ATR) and DNA-dependent protein kinase catalytic subunit (DNA-PK). These enzymes detect damage and activate the appropriate DSB repair pathways, namely homologous recombination or non-homologous end joining to maintain genomic integrity. Targeting these major DNA damage response kinases is a promising therapeutic target in cancers, including UM, to enhance the therapeutic effect of radiation and potentially reduce the radiation dosage required for treatment, thus reducing the frequency and severity of adverse effects.

**Methods:**

Here, we investigate the response of 3D spheroid models of UM to both X-rays and PBT in the presence of inhibitors of ATM (AZD1390), ATR (AZD6738) and DNA-PK (AZD7648).

**Results:**

We demonstrate that inhibition of these protein kinases significantly suppresses the growth of UM spheroids exposed to both X-rays and PBT, which is primarily driven by delayed and persistent DSBs, leading to the accumulation of chromosomal aberrations.

**Discussion:**

These results highlight the potential of combining ATM, ATR or DNA-PK inhibitors to enhance radiotherapy efficacy, and particularly with targeted PBT, to help improve clinical outcomes in UM.

## 1 Introduction

Uveal Melanoma (UM) is a rare but aggressive primary intraocular malignancy in adults, with an incidence rate of 5–8 cases per million (1, 2). An estimated 50% of patients will progress to develop metastases to the liver, resulting in a poor median survival of 4–7 months (2–4). In the UK, localised radiotherapy, such as plaque brachytherapy or proton beam therapy (PBT), is used to treat the primary tumour in most patients, with surgical resection of the eye (enucleation) performed in approximately 30% of cases (5, 6). PBT is effective at controlling the primary tumour and maintaining high eye preservation rates, however, a small subset of patients can experience local tumour recurrence (7–9). Side effects of PBT include radiation retinopathy and secondary neovascular glaucoma, which can decrease vision and impact quality of life (9–11). Despite the importance of radiotherapy for primary UM treatment, the radiobiology of the tumour response, and particularly the efficiency of signalling the DNA damage, is surprisingly underexplored.

The therapeutic response of ionising radiation, including X-rays/photons and PBT, is largely caused by the induction of extensive DNA damage within the tumour cells, which exceeds their DNA repair thresholds and encourages either apoptotic or mitotic cell death (12). The most critical lesions driving this effect are DNA double strand breaks (DSBs) and complex DNA damage (CDD), consisting of multiple DNA lesions within close proximity to each other, due to their more difficult nature of repair compared to isolated DNA damage (13, 14). Following DNA damage induction, the key protein kinases that orchestrate and co-ordinate the cellular response to DNA DSBs are ataxia telangiectasia mutated (ATM), ataxia telangiectasia and Rad3 related (ATR) and DNA-dependent protein kinase (DNA-PK) (15, 16). These proteins largely activate distinct DSB repair pathways, with ATM and DNA-PK primarily enabling non-homologous end-joining (NHEJ), and ATR-promoting homologous recombination (HR). Due to this, targeting either ATM, ATR or DNA-PK with specific inhibitors has been proposed as a strategy to enhance the biological effect of ionising radiation by preventing accurate and timely DNA repair. This approach has been shown to be successful in improving the response to X-rays or PBT in a variety of cancer types, such as head and neck, glioblastomas, colorectal, and cervical carcinomas amongst many others (17–20). However, this strategy has not yet been fully explored in UM, and which was highlighted in our recent review of the field (12).

Discovery of a radiosensitisation agent in UM could allow a lower radiation dose to be used in clinical treatments, and therefore potentially reduce adverse treatment effects. This could also additionally lead to alternative treatment options for larger tumours currently treated with enucleation, whilst protecting the other delicate cells and structures within the eye. Interestingly, DNA-PK has been shown to have higher gene expression in metastatic UM and is associated with poor survival, therefore, making this kinase a particularly appealing target for radiosensitisation (21). Indeed, it has been suggested that the activity of DNA-PK and of NHEJ is upregulated in UM, and that UM cell lines were specifically sensitive to inhibitors for DNA-PK (NU7026 and NU7441) both alone and in the presence of X-rays (22). NU7441 has also been shown to sensitise UM cells to photon radiation, particularly those that have acquired radioresistance through repeated radiation exposure where enhanced DNA-PK activation was shown (23). Previous data has furthermore also shown that different UM cell lines show altered expression of ATM, and that a previous generation ATM inhibitor (KU-55933) could sensitise UM cell lines to both X-rays and PBT (6). Despite this, there is still a lack of supporting evidence of the efficiency of UM cells to perform DSB repair, in response to both photon and proton radiation, as well as a lack of discovery of the optimal DNA repair inhibitors that synergise with the radiation in effective cell killing. It is also worth noting that the use of 3D models for UM that more accurately recapitulate some of the physical features of the tumour, including oxygen and nutrient gradients, and their response to PBT or X-rays is less well understood.

By using potent and specific inhibitors of ATM, DNA-PK and ATR, we aimed to discover whether this can potentiate and exacerbate the response to radiation in 3D spheroid models of UM.

## 2 Methods

### 2.1 Inhibitors and antibodies

Inhibitors for ATM (AZD1390), ATR (AZD6738), and DNA-PK (AZD7648) were kindly provided by AstraZeneca UK. Antibodies against γH2AX (05-636; Merck-Millipore, Watford, UK), RAD51 (ab133534; Abcam, Cambridge, UK), 53BP1 (A300-272A; Bethyl Labs, Montgomery, Texas, USA) and mitosin (6107688; BD Transduction Laboratories, Poland) were used for immunofluorescent staining, along with goat anti-mouse Alexa Fluor 555 and goat anti-rabbit Alexa Fluor 488 (Life Technologies, Paisley UK) secondary antibodies.

For immunoblotting the following antibodies were used: phosphorylated (S1981) ATM (ab81292), ATM (ab78), ATR (ab2905) and phosphorylated (S2056) DNA-PK catalytic subunit (ab18192), were all from Abcam (Cambridge, UK). DNA-PK (sc-9051) was from Santa Cruz Biotechnology (Heidelberg, Germany), phosphorylated (T1518) ATR was from Cell Signalling Technology (Massachusetts, USA) and tubulin (T6199) were from Merck-Millipore (Watford, UK). Goat anti-mouse Alexa Fluor 680 or goat anti-rabbit Alexa Fluor 800 secondary antibodies were from Life Technologies (Paisley, UK).

### 2.2 Radiation sources

X-ray irradiations were performed at a dose rate of ~3 Gy/min using a 150 kV CellRad+ X-ray irradiator (Faxitron Bioptics, Tucson, AZ, USA). PBT irradiations were performed using the MC-40 cyclotron utilising a 28 MeV beam at a dose rate of ~5 Gy/min, as previously described (24).

### 2.3 Cell and spheroid culture, growth and viability assays

All immortalised UM cell lines (92.1, Mel202, Mel270, MP41, MP46, OMM1 and OMM2.3) were routinely cultured as monolayers in RPMI1640 (Merck-Millipore, Watford, UK) supplemented with 10% foetal bovine serum, 1x non-essential amino acids and 1x penicillin streptomycin in 5% CO_2_ at 37 °C.

Cells were seeded at 500 cells per well in triplicate in 100 µl complete media in 96-well ultra-low attachment plates (Corning B.V. Life Sciences, Amsterdam, The Netherlands) and left for 48 h to form spheroids of ~400-800 µM in diameter. The spheroids were incubated with either ATM, ATR or DNA-PK inhibitors, or with DMSO as the vehicle control, for 1 h. Following irradiation, spheroids received two 50% media changes. Spheroid growth was monitored up to day 18 post-seeding, during which time images were captured using the EVOS M5000 Imaging system (Life Technologies, Paisley, UK). Image J was used to threshold the image and calculate the cross-sectional spheroid area in µm².

For viability assays, at 7 days post-irradiation the spheroids were transferred to black walled 96 well plates (Corning B.V. Life Sciences, Amsterdam, The Netherlands), and viability performed using CellTiter-Glo 3D as detailed by the manufacturer (Promega, Wisconsin, USA). Hydrogen peroxide (10 mM for 15 min) was used as a positive control. All conditions were normalised against the unirradiated, DMSO-treated spheroids and viability calculated relative to the positive control.

### 2.4 Immunoblotting

Spheroids were harvested at the specific time points post-irradiation and pelleted by centrifugation (300 x *g* at 4 °C for 5 min). The pellets were frozen overnight at -40 °C, then resuspended in RIPA buffer (20 mM Tris-HCl pH 7.8, 150 mM NaCl, 2.5 mM EDTA, 1% Glycerol, 0.1% SDS, 0.5% SDOC, 1% NP40) containing 1 µg/ml protease inhibitors (pepstatin A, aprotinin, chymostatin, leupeptin), 1 mM DTT, 0.1 mM PMSF and 10 μg/ml phosphatase cocktail (all Merck-Millipore, Watford, UK). Cells were incubated by rotation for 30 min at 4 °C, then centrifuged (14,000 x *g*, 20 min at 4 °C) and the supernatant collected.

Total protein (30 μg) was separated on 4-12% Tris-glycine SDS gradient gels (Thermo Fisher Scientific, Waltham, USA), and proteins transferred onto an Immobilon FL PVDF membrane (Merck-Millipore, Watford, UK). Membranes were blocked using Odyssey blocking buffer (Li-cor Biosciences, Cambridge, UK) and incubated with the primary antibody overnight at 4 °C. Membranes were washed with PBS containing 0.1% Tween 20, incubated with either Alexa Fluor 680 or IR Dye 800 secondary antibodies (Thermo Fisher Scientific, Waltham, USA) for 1 h at room temperature and further washed with PBS containing 0.1% Tween 20. Proteins were then visualised and quantified using the Odyssey image analysis system (Li-cor Biosciences, Cambridge, UK).

### 2.5 Neutral comet assay

Following irradiation (4 Gy), spheroids were left to repair for 0–4 h, then 20 spheroids per condition were collected by centrifugation (1,500 x *g* for 5 min at 4 °C), washed with PBS, resuspended in accutase and incubated until a single cell suspension was generated (~3 min). The cell suspension was embedded in 1% low melting point agarose and added onto normal melting point agarose-precoated microscope slides and allowed to set on ice for ~3 min. The slides were then lysed for at least 1 h in cold lysis buffer (2.5 M NaCl, 100 mM EDTA disodium salt, 10 mM Tris base, 1% N-lauroylsarcosine, 1% DMSO, and 1% Triton X-100; pH 9.5). Slides were then placed in cold electrophoresis buffer (1x TBE, pH 8.3) for 30 min to enable DNA unwinding, prior to electrophoresis at 25 V, ~15 mA for 25 min. The slides were then washed twice with cold PBS before being allowed to dry overnight. The agarose was rehydrated with water (pH 8.0) for 30 min before the DNA was stained with SYBR gold (Merck-Millipore, Watford, UK) diluted 1:10,000 in water (pH 8.0) for 30 min. The SYBR Gold was removed and the slides left to dry overnight. Comets were visualised using an Olympus BX53 fluorescent microscope with a 10x objective, images were captured and analysed using the Komet 6.0 image analysis software (Andor Technology, Belfast, Northern Ireland) to determine % tail DNA from at least 50 cells per replicate. Two slides per condition and three individual biological experiments were performed.

### 2.6 Immunofluorescence

Following irradiation, spheroids were harvested by centrifugation (300 x *g* for 5 min at 4 °C), washed with PBS, resuspended in accutase and incubated until a single cell suspension was generated (~3 min). The cells were quenched with media and centrifuged onto slides at 1000 x *g* for 5 min with a cytospin (Thermo Scientific Cytospin 4 Cytocentrifuge). The cells were fixed with 10% neutral buffered formalin for 10 min, permeabilised with 0.3% Triton X-100 in PBS for 10 min, washed three times with PBS, and then blocked with 2% BSA in PBS for 1 h. γH2AX, 53BP1 or Rad51 and mitosin antibodies in 2% BSA were added and the slides placed in humidified chambers at 4 °C overnight. After three washes with 0.1% Tween-20 in PBS, the slides were incubated at room temperature in the dark with Alexa Fluor 555 or Alexa Fluor 488 secondary antibodies in 2% BSA for 1 h. The slides were then washed three times with 0.1% Tween-20 in PBS, before a coverslip was placed on top of the cells with fluoroshield containing DAPI (Merck-Millipore, Watford, UK). The cells were visualised using an Olympus BX53 fluorescent microscope with a 60x objective. At least 100 cells per replicate were analysed and foci counted using GUI Foco software. Two slides per condition and three individual biological experiments were performed.

### 2.7 Cytokinesis-block micronucleus assay

Following irradiation, cytochalasin B (1μg/ml) was added to the spheroids to block the first cytokinesis process at a cell line-specific time:- 24 h for 92.1, 32 h for OMM2.3. Spheroids were harvested by centrifugation (300 x *g* for 5 min at 4 °C), and then dissociated by resuspending in accutase for 3 min at 37 °C. The single cell suspension was treated with a hypotonic solution (75 mM KCl) for 3 min, with a 3:5 methanol: acetic acid solution and then washed once with methanol and subsequently fixed twice with 6:1 methanol: acetic acid. The cell solution was then added to clean cold glass slides and left to dry overnight. Staining was performed by immersing the slides in 2% Giemsa solution in Sorensen’s buffer (pH 6.8) for 10 min.

The criteria for scoring was followed (25). In brief, for analysis of cell proliferation, 1000 cells were analysed to determine the ratio of mono- (MN), bi- (BN) and multinucleated cells (MuN) per treatment, and which was used to calculate the cytochalasin B proliferation index (CBPI). The frequency of binucleated cells containing one or more micronuclei per 1000 binucleated cells was scored, along with other damage events in once divided binucleated cells per 1000 cells, namely nucleoplasmic bridges and nuclear buds.

### 2.8 Cell cycle analysis

Spheroids were harvested 8 and 24 h post-irradiation by centrifugation (300 x *g* for 5 min at 4 °C) before being resuspended in accutase to generate a single cell suspension. The cells were fixed in 70% ethanol for at least 1 h, then stained with propidium iodide (10 µg/ml) with RNAse A (0.1 mg/ml) in PBS with 0.05% Tween-20 for 1 h. A CytoFlex was used for cytometry analysis [Beckman Coulter Life Sciences, Indianapolis, USA], and the data was analysed by Floreida.io.

### 2.9 Statistical analysis

All experiments were performed in triplicate as independent, biological experiments. Dose enhancement ratios (DER) were calculated from relative spheroid growth curves (from days 3–12 relative to the radiation dose used), at a dose required to reduce growth by 25%, comparing the inhibitor treatment to the DMSO-treated spheroids. Statistical analysis of DNA damage and repair (γH2AX, 53BP1, RAD51 foci, cytokinesis blocked micronuclei, and comet assays) and cell cycle data was performed using either a one or two sample *t* test.

## 3 Results

### 3.1 ATM, DNA-PK and ATR inhibition sensitises UM spheroids to X-ray radiation

**Figure 1:**
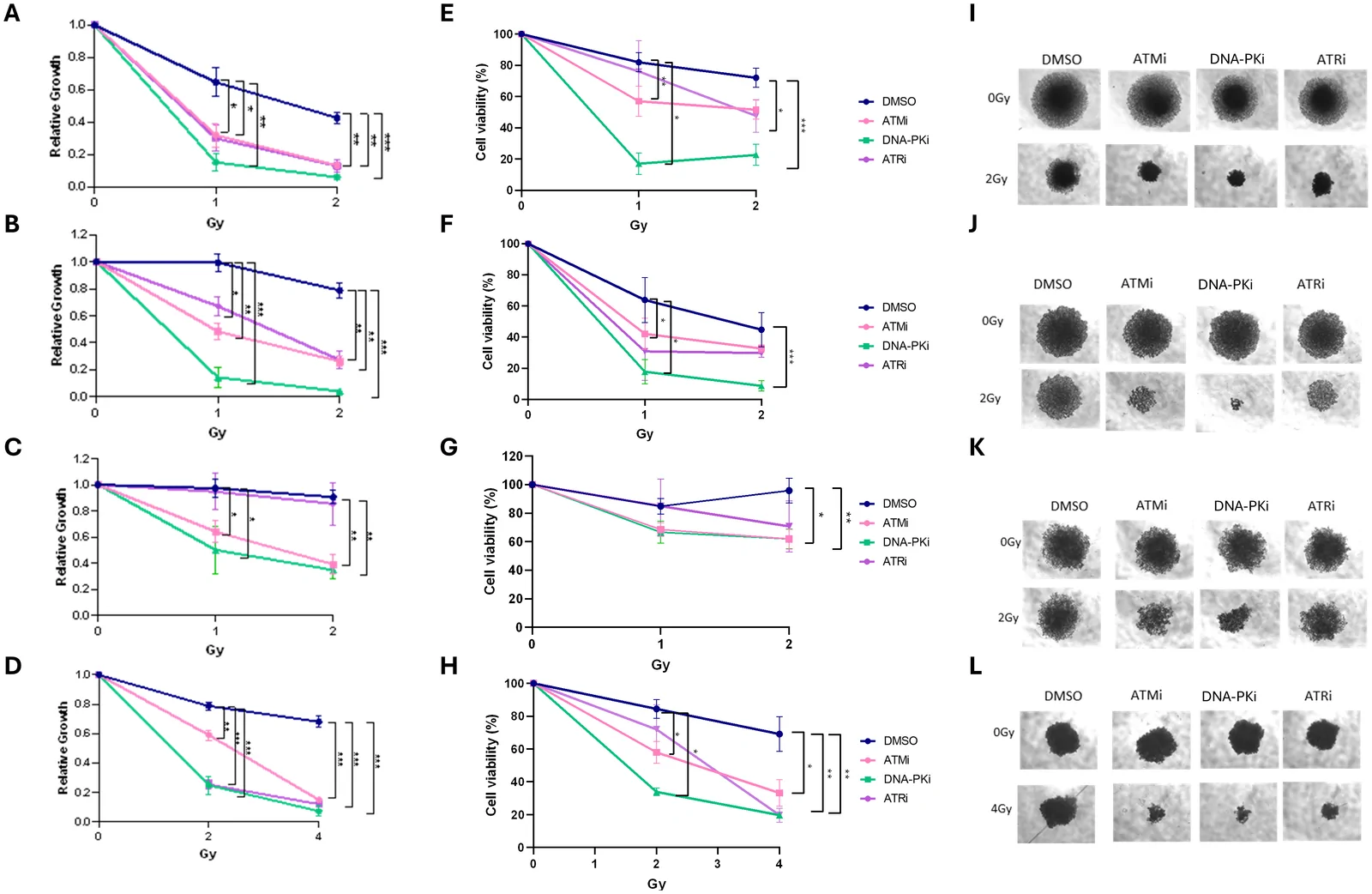
ATM, DNA-PK and ATR inhibition in combination with X-rays supresses growth and viability of UM spheroid models. Spheroids of **(A, E)** 92.1, **(B, F)** Mel202, **(C, G)** MP41 and **(D, H)** OMM2.3 were treated with ATM inhibitor (AZD1390; 0.2 μM), DNA-PK inhibitor (AZD7648; 1 µM) or ATR inhibitor (AZD6738; 1 μM for 92.1 or 2 μM for MP41/OMM2.3) for 1 h prior to X-ray irradiation (1 or 2 Gy). **(A–D)** Relative change in spheroid growth was assessed from measuring the change in area from day 3 to day 15 post-seeding, and normalised against the unirradiated control which was set to 1.0. **(E–H)** Cell viability was evaluated 7 days post-irradiation. **(I–L)** Representative images of spheroids at day 15 post-seeding comparing irradiated (2 Gy) versus unirradiated conditions across treatment groups. Spheroids were seeded in triplicate, with data analysed from three biologically independent experiments and expressed as mean ± SD. *p<0.02, **p<0.01, ***p<0.005 as analysed by a two-sample *t* test.

UM cell lines (7 models in total) were grown as 3D spheroids to more accurately replicate the structure of the original tumour and how this responds to radiotherapy. Spheroids were allowed to form and grow for 48 h prior to a single dose of X-rays (1–4 Gy). Spheroid growth was monitored between days 3–12 post-seeding (**Supplementary Figures 1A–I**), and data relative to radiation dose normalised against the unirradiated, DMSO-treated spheroids (**Figures 1A–D, I–L**; **Supplementary Figures 2A, D, G, C, F, I**). It was noticeable that there was a marked variation in the sensitivity of the UM models to X-rays, where for example, 92.1 spheroids were the most radiosensitive as shown by a 60% reduction in growth following 2 Gy irradiation, whereas MP41 and OMM2.3 exhibited only a 10-20% reduction in growth following the same treatment (**Figures 1A, I, C, D, K, L**, respectively). These data were also replicated through monitoring cell viability (**Figures 1E–H**; **Supplementary Figures 2B, E, H**), using hydrogen peroxide (10 mM) as an appropriate positive control (**Supplementary Figure 3**).

The impact of ATM, DNA-PK and ATR inhibition on spheroid growth and viability was then assessed to determine the impact on UM radiosensitisation. The inhibitors were used at concentrations of 0.2 μM for the ATM inhibitor (AZD1390), and 1 μM for both the DNA-PK (AZD7648) and ATR (AZD6738) inhibitors. Exceptions included the OMM1, OMM2.3, and MP41 cell lines, which received 2 μM AZD6738 as this concentration provided a clearer inhibitory effect in these models, whilst remaining below the threshold of toxicity. Effectiveness of the inhibitors in suppressing radiation-induced activation of the kinases was confirmed by immunoblotting, specifically through inhibition of site-specific phosphorylation sites within ATM (S1981), DNA-PK catalytic subunit (S2056) and ATR (AZD6738). Notably, using 92.1 and OMM1 cell lines, phosphorylation of ATM and DNA-PK catalytic subunit was suppressed by ~80-90% by the respective inhibitors, whereas ATR phosphorylation was only suppressed by ~50% (**Supplementary Figures 4A–C**) the dose of which could not be increased due to significant toxicity observed in completely suppressing spheroid growth. Indeed, in the majority of cell lines, the inhibitors alone at the concentrations used were relatively non-toxic, although significant reduction of spheroid growth was seen with the ATR inhibitor in Mel202, Mel270, MP41 and MP46 cells and of DNA-PK inhibitor on MP46 cells (**Supplementary Figures 5A–N**).

When combined with X-ray radiation, the inhibitors consistently suppressed spheroid growth and viability, highlighting their radiosensitising capabilities (**Figures 1A–L**; **Supplementary Figures 2A–I**). Among the three, DNA-PK inhibition produced the most pronounced effects across all seven UM spheroid models, as revealed by DERs ranging from 2.5-10.0 (**Table 1**). The second most effective inhibitor was ATM (DER = 1.6-8.0), whereas ATR was the least effective radiosensitiser (DER = 1.3-5.6). The degree of radiosensitivity enhancement was cell line dependent and based on inherent sensitivity. For example, 92.1 as the most radiosensitive model displayed low radiosensitisation with all three inhibitors (DER = 1.9-2.5), whereas Mel202 and OMM2.3 displayed higher radiosensitisation (DER = 3.2-9.5 and 2.3-5.6, respectively). Based on these findings, 92.1 and OMM2.3 spheroids were selected for further investigation into validating the mechanism of action of the inhibitors related to effects on DNA damage and repair.

**Table 1:** Radiosensitisation of UM spheroid models in response to X-rays.

Inhibitor	92.1	Mel202	Mel270	MP41	MP46	OMM1	OMM2.3
ATM	1.9	4.8	3.0	8.0	1.6	2.7	2.3
DNA-PK	2.5	9.5	4.8	10.0	3.2	5.0	5.6
ATR	1.9	3.2	4.8	1.4	1.3	2.2	5.6

Dose Enhancement Ratios (DER) for each drug-radiation combination were calculated at 25% spheroid growth inhibition.

### 3.2 ATM, DNA-PK and ATR inhibition with X-ray irradiation causes persistent DNA DSBs

**Figure 2:**
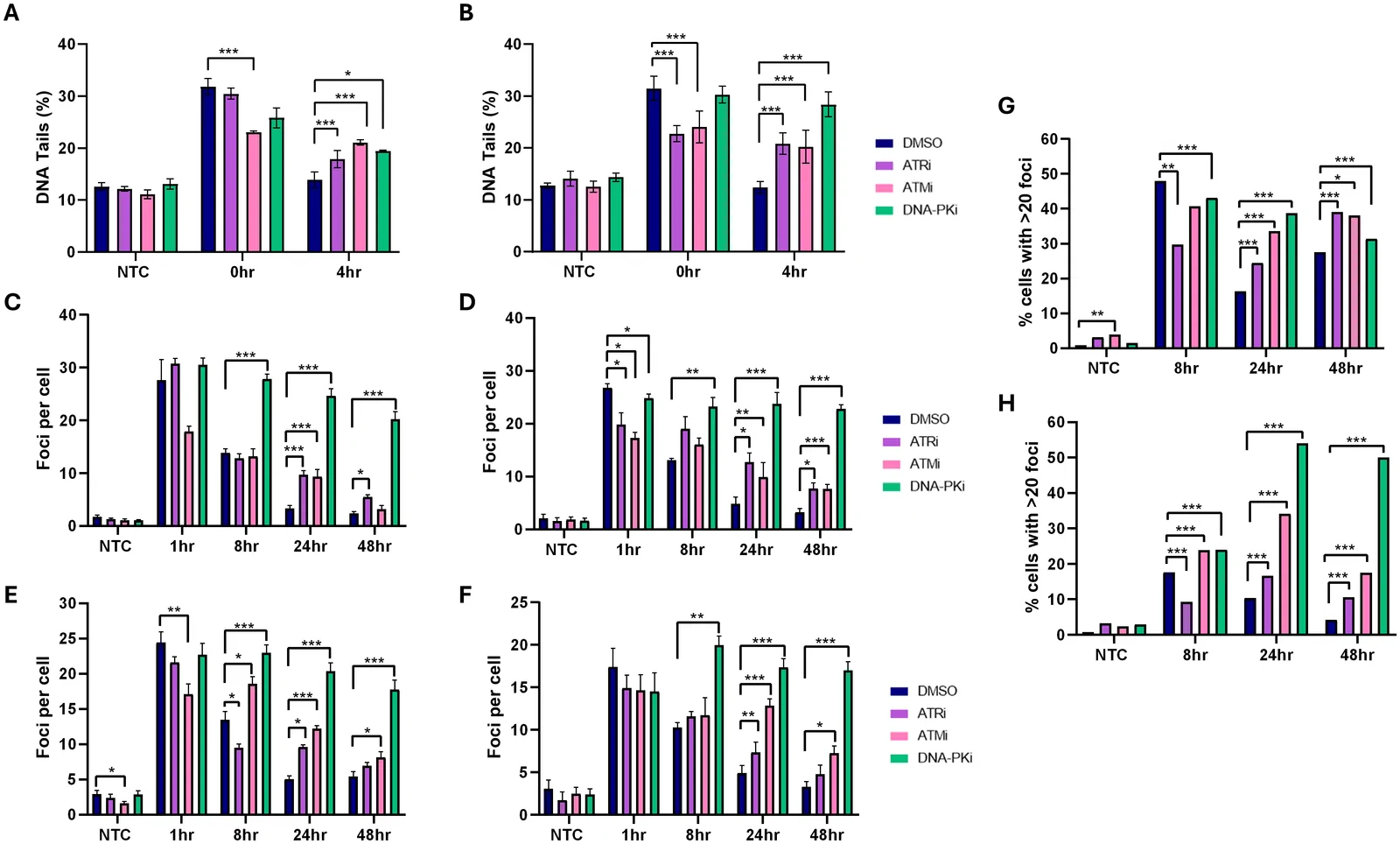
ATM, DNA-PK and ATR inhibition cause persistence of X-ray-induced DNA DSBs. **(A, C, E, G)** 92.1 and **(B, D, F, H)** OMM2.3 spheroids were treated with ATM inhibitor (AZD1390; 0.2 μM), DNA-PK inhibitor (AZD7648; 1 µM) or ATR inhibitor (AZD6738; 1 μM for 92.1 or 2 μM for OMM2.3) for 1 h prior to X-ray irradiation (4 Gy). **(A–B)** DNA DSBs were measured using the neutral comet assay, and data is expressed as % DNA tail. **(C–D)** γH2AX, **(E–F)** 53BP1 or **(G–H)** Rad51 foci were determined using immunofluorescence microscopy. Rad51 foci data is expressed as the percent of cells with >20 foci. All experiments are shown as the mean ± SD of three biologically independent experiments. *p<0.05, **p<0.01, ***p<0.001, as analysed by a one-sample t test.

Using the neutral comet assay to directly assess double-strand breaks (DSBs) in 92.1 and OMM2.3 spheroids at a 4 Gy X-ray dose that achieved a sufficiently measurable level of the DNA damage, we observed that all three inhibitors significantly impaired the complete resolution of the damage 4 h post X-ray irradiation, in comparison to the DMSO treated spheroids where almost complete repair of radiation-induced DSBs was observed (**Figures 2A, B**; **Supplementary Figures 6A, B**). Interestingly, inhibition of ATM was shown to reduce the levels of DSBs immediately post-irradiation, whereas all the inhibitors had no impact on the levels of DSBs in the absence of X-ray irradiation. To compliment these data, we also analysed γH2AX and 53BP1 foci as surrogate markers of DSBs at a 4 Gy X-ray dose for direct comparison with the comet assay data. In both spheroid models, inhibition of DNA-PK had the most striking impact on suppressing resolution of γH2AX and 53BP1 foci from 8–24 h post X-ray irradiation (**Figures 2C–F**; **Supplementary Figures 7A–D**). However, ATM and ATR inhibition also led to delayed resolution of γH2AX and 53BP1 foci, with levels persisting for at least 8–24 h post-X-ray irradiation. Notable is that ATM inhibition caused a marked reduction in the levels of γH2AX foci at 1 h post-irradiation, supporting that this is the major kinase involved in phosphorylation of H2AX in UM cells and in triggering the signalling response to DSBs.

Next, the proficiency of HR was investigated using Rad51 foci in mitotically active (mitosin positive) cells, again following a 4 Gy X-ray dose. The percentage of cells with >20 foci were scored to categorise cells with moderate to high levels of HR. Interestingly, there was noticeable differences between the two spheroid models, with OMM2.3 cells displaying that ~20% of cells at 8 h post-irradiation contained >20 foci whereas this was significantly higher (~50%) in 92.1 cells, likely associated with proliferative rates (**Figures 2G, H**; **Supplementary Figures 8A–D**). Nevertheless, similar trends were observed with ATM and ATR inhibitors in both models, leading to delays in resolution of RAD51 foci at 24 and 48 h post-radiation compared to DMSO-treated spheroids. Striking differences were observed between the 92.1 and OMM2.3 cells in response to DNA-PK inhibition, which resulted in similar levels of cells with persistent RAD51 foci in 92.1 cells, whereas in OMM2.3 these almost doubled at 24 and 48 h post-irradiation. Collectively, these results demonstrate that inhibition of ATM, DNA-PK and ATR disrupt DSB signalling and repair kinetics, which contributes to the enhanced radiosensitivity.

### 3.3 Inhibition of ATM, DNA-PK and ATR enhances X-ray-induced chromosomal aberrations and alters cell cycle progression

**Figure 3:**
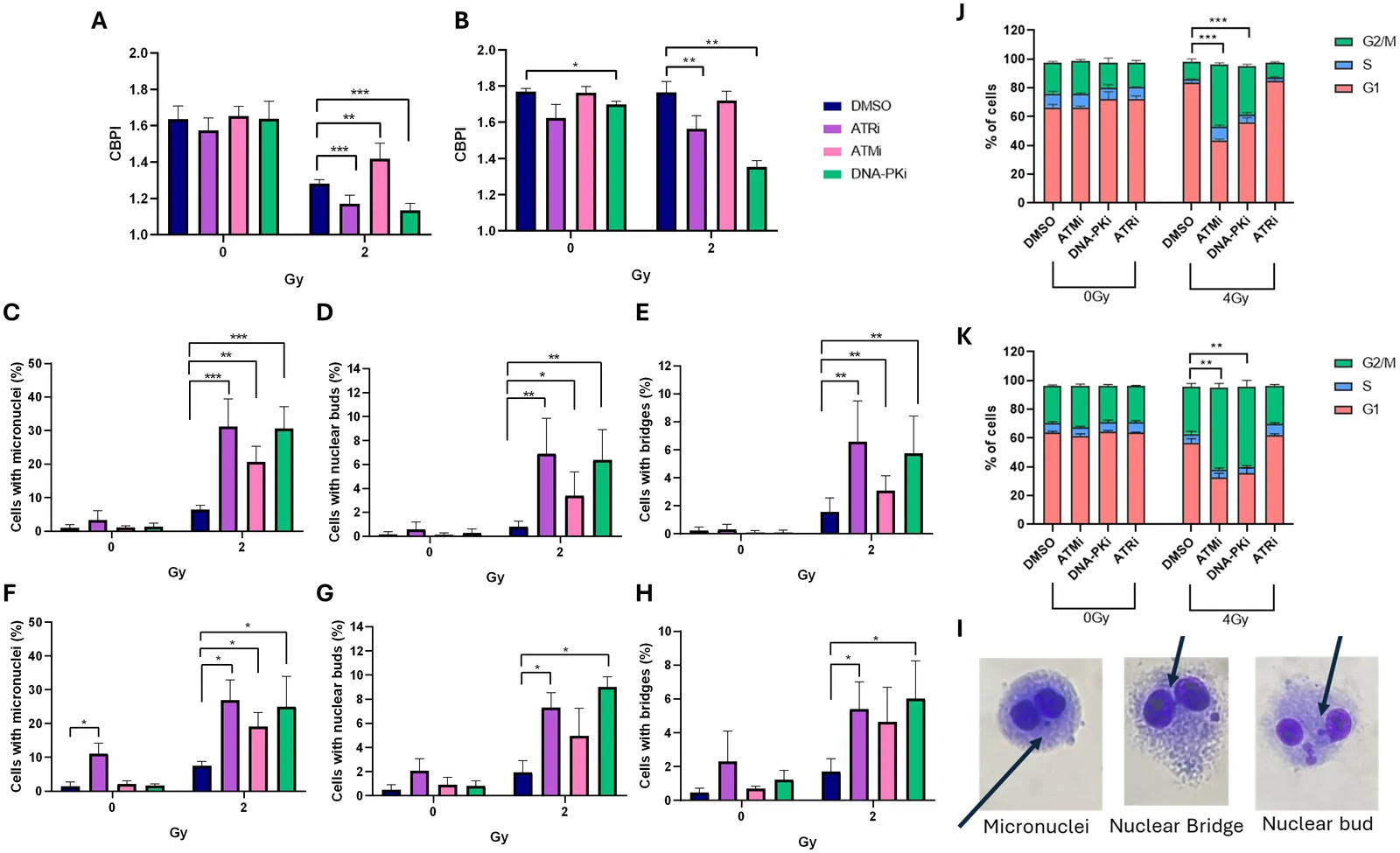
ATM, DNA-PK and ATR inhibition in combination with X-rays causes chromosomal aberrations and altered cell cycle progression. **(A, C–E, J)** 92.1 and **(B, F–H, K)** OMM2.3 spheroids were treated with ATM inhibitor (AZD1390; 0.2 μM), DNA-PK inhibitor (AZD7648; 1 µM) or ATR inhibitor (AZD6738; 1 μM for 92.1 or 2 μM for OMM2.3) for 1 h prior to X-ray irradiation (4 Gy). **(A–B)** Cytokinesis-block proliferation index (CBPI), as well as the percentage of cells (± SD) with **(C, F)** micronuclei, **(D, G)** nuclear buds and **(E, H)** nucleoplasmic bridges were scored in 1000 cells. **(I)** Representative images of the different nucleated cells, and the chromosomal aberrations scored. **(J–K)** Cell cycle analysis was performed by flow cytometry, represented as mean ± SD number of cells in each cell cycle phase. *p<0.05, **p<0.01, ***p<0.005 as analysed by a one-sample t test.

We next investigated whether DSB repair inhibition in combination with X-ray irradiation impacts chromosomal stability and cell cycle dynamics in UM spheroids. The proliferation index, measured by the CBPI, decreased in 92.1 spheroids following X-ray treatment alone (at a 2 Gy dose to ensure accurate measurement of chromosomal aberrations), whereas in OMM2.3 spheroids this was less pronounced (**Figures 3A,B**), likely reflecting the inherent radioresistance of this model. Inhibition of DNA-PK and ATR when combined with X-rays, led to a further significant reduction in the CBPI compared to the respective DMSO-treated spheroids. Interestingly, ATM inhibition post-irradiation led to a significantly higher CBPI in 92.1 spheroids compared to the DMSO-irradiated models, which was not observed in OMM2.3 spheroids. Analysis of micronuclei frequency showed that this increased significantly in both 92.1 and OMM2.3 spheroids with all three inhibitor-radiation combinations compared to the respective DMSO-treated controls (**Figures 3C, F**). Chromosomal rearrangements, indicated by nuclear buds and nucleoplasmic bridges (**Figures 3D, E, G–I**), were also significantly increased with ATR and DNA-PK inhibition following X-ray irradiation in both models versus the DMSO-irradiated controls, whereas following ATM inhibition with X-rays increased chromosome rearrangements were seen but which was only statistically significant in 92.1 spheroids. Taken together, these findings indicate that inhibition of ATM, ATR or DNA-PK significantly enhances chromosomal aberrations following X-ray irradiation in UM spheroids.

Cell cycle analysis revealed distinct effects of ATM, DNA-PK, and ATR inhibition following X-ray irradiation (at a 4 Gy dose comparable to the DSB measurements). ATM and DNA-PK inhibition resulted in a significantly elevated G2/M population at 24 h post-irradiation in both spheroid models versus the DMSO-irradiated controls (**Figures 3J, K**; **Supplementary Figures 9A–H**), indicating a prolonged arrest and delayed cell cycle recovery. In contrast, ATR inhibition post-irradiation had no significant impact on G2/M arrest versus the DMSO-irradiated spheroids.

### 3.4 ATM, DNA-PK and ATR inhibition sensitises UM spheroids to PBT

**Figure 4:**
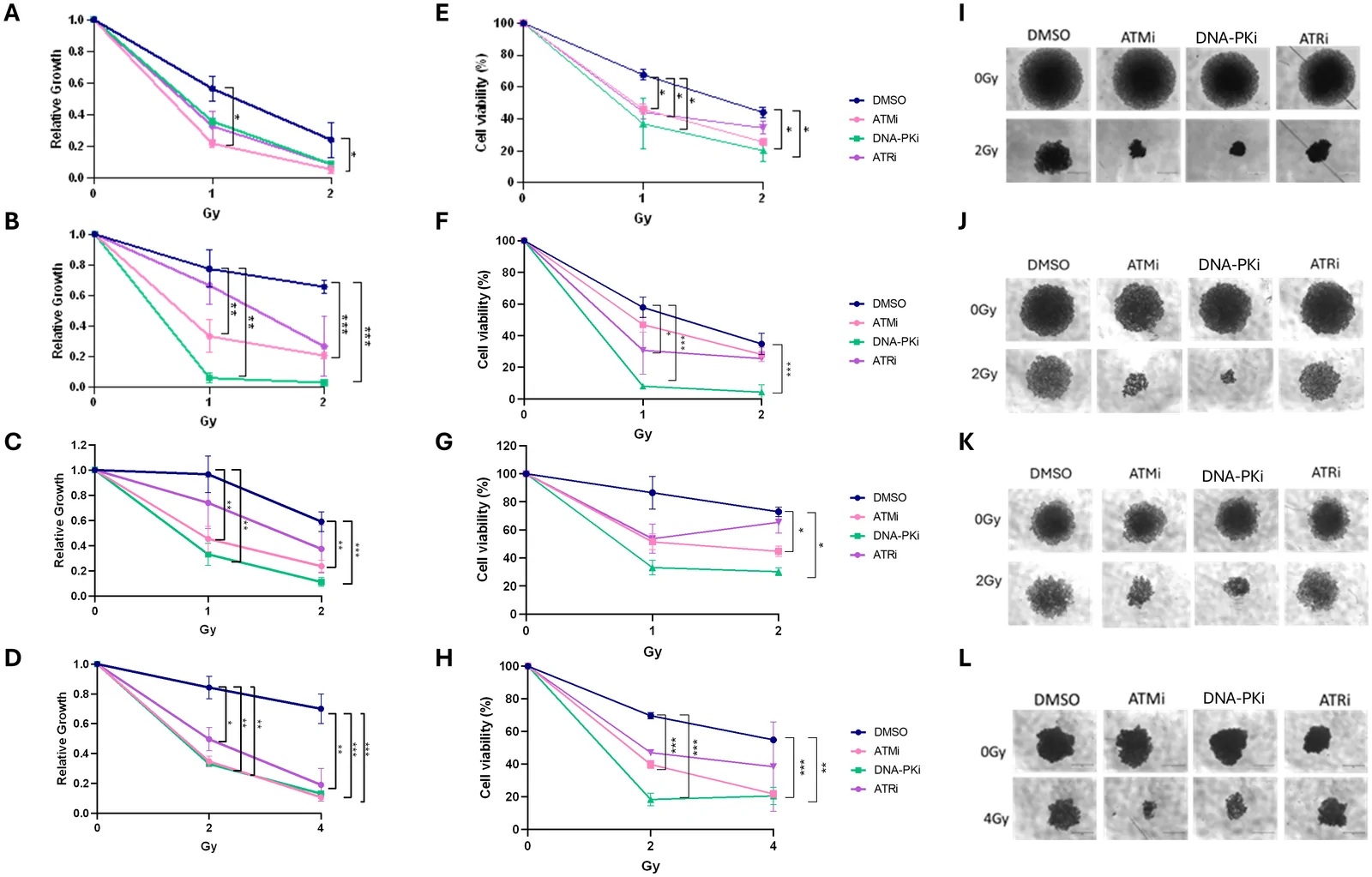
ATM, DNA-PK and ATR inhibition in combination with PBT supresses growth and viability of UM spheroid models. Spheroids of **(A, E)** 92.1, **(B, F)** Mel202, **(C, G)** MP41 and **(D, H)** OMM2.3 were treated with ATM inhibitor (AZD1390; 0.2 μM), DNA-PK inhibitor (AZD7648; 1 µM) or ATR inhibitor (AZD6738; 1 μM for 92.1 or 2 μM for MP41/OMM2.3) for 1 h prior to PBT (1 or 2 Gy). **(A–D)** Relative change in spheroid growth was assessed from measuring the change in area from day 3 to day 15 post-seeding, and normalised against the unirradiated control which was set to 1.0. **(E–H)** Cell viability was evaluated 7 days post-irradiation. **(I–L)** Representative images of spheroids at day 15 post-seeding comparing irradiated (2 Gy) versus unirradiated conditions across treatment groups. Spheroids were seeded in triplicate, with data analysed from three biologically independent experiments and expressed as mean ± SD. *p<0.02, **p<0.01, ***p<0.005 as analysed by a two-sample t test.

Following responses to X-ray irradiation, the seven UM spheroid models were also irradiated with targeted PBT (1–4 Gy) and growth curves established (**Supplementary Figures 10A–I**). Following normalisation relative to the unirradiated control, inhibition of DNA-PK and ATM appeared to be the most potent radiosensitising agent with PBT through monitoring spheroid growth (**Figures 4A–D**; **Supplementary Figures 11A, C, D, F, G, I**), with DER values of 1.7-5.6 and 1.9-5.6, respectively (**Table 2**). Inhibition of ATR appeared to show the weakest radiosensitisation, as revealed with DER values of 1.5-3.5. Similar to experiments using X-rays, the degree of radiosensitivity enhancement with the inhibitors following proton irradiation was cell line dependent. The 92.1 model again showed low radiosensitisation with all three inhibitors (DER = 1.7-2.0), whereas Mel202 and OMM2.3 displayed higher radiosensitisation (DER = 1.5-5.0 and 3.2-5.3, respectively). Effects on spheroid growth also correlated with the impact of the inhibitor-proton combinations on cell viability (**Figures 4E–H**; **Supplementary Figures 11B, E, H**).

**Table 2:** Radiosensitisation of UM spheroid models in response to PBT.

Inhibitor	92.1	Mel202	Mel270	MP41	MP46	OMM1	OMM2.3
ATM	2.0	3.3	5.6	4.3	1.9	4.0	5.3
DNA-PK	1.7	5.0	5.6	5.2	2.0	2.5	5.3
ATR	1.7	1.5	3.5	1.6	3.0	1.5	3.2

Dose Enhancement Ratios (DER) for each drug-radiation combination were calculated at 25% spheroid growth inhibition.

### 3.5 ATM, DNA-PK and ATR inhibition with proton irradiation causes persistent DNA DSBs

**Figure 5:**
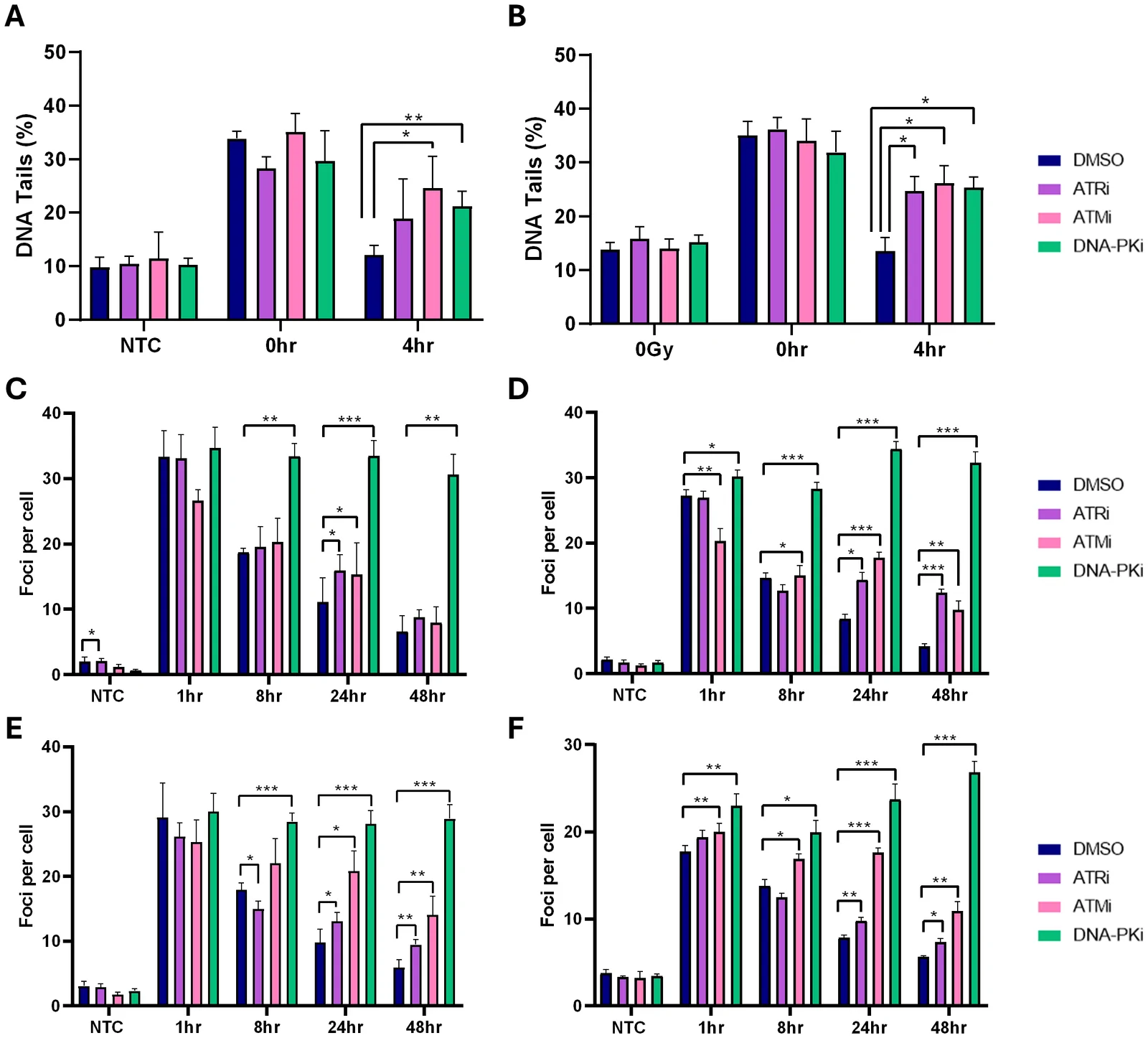
ATM, DNA-PK and ATR inhibition cause persistence of PBT-induced DNA DSBs. **(A, C, E)** 92.1 and **(B, D, F)** OMM2.3 spheroids were treated with ATM inhibitor (AZD1390; 0.2 μM), DNA-PK inhibitor (AZD7648; 1 µM) or ATR inhibitor (AZD6738; 1 μM for 92.1 or 2 μM for OMM2.3) for 1 h prior to PBT (4 Gy). **(A–B)** DNA DSBs were measured using the neutral comet assay, and data is expressed as % DNA tail. **(C–D)** γH2AX or **(E–F)** 53BP1 foci were determined using immunofluorescence microscopy. All experiments are shown as the mean ± SD of three biologically independent experiments. *p<0.05, **p<0.01, ***p<0.001, as analysed by a one-sample t test.

The effects of ATM, DNA-PK and ATR inhibition on DSB induction and repair following PBT (4 Gy) in spheroid models of 92.1 and OMM2.3 were investigated using neutral comet assays and immunofluorescence microscopy for γH2AX and 53BP1 foci. Analysing DSBs directly using the neutral comet assay revealed that there was significant persistence of the damage 4 h post radiation following both ATM and DNA-PK inhibition in both 92.1 and OMM2.3, compared to the DMSO-irradiated controls where the majority of DSBs had been repaired (**Figures 5A, B**; **Supplementary Figures 12A, B**). Whereas with ATR inhibition, DSBs were elevated at 4 h post-PBT in both models but was only statistically significant in OMM2.3. On analysis of γH2AX foci, DNA-PK inhibition was again the most potent at almost completely preventing resolution of PBT-induced DSB signalling up to 24 h post-irradiation in both 92.1 and OMM2.3 spheroids compared to DMSO-irradiated spheroids (**Figures 5C, D**; **Supplementary Figures 13A, B**). Both inhibition of ATM and ATR significantly delayed DSB signalling and resolution, which was statistically significant at the 24 h time point compared to DMSO-irradiated spheroids in both cell models. Similar data was revealed using 53BP1 foci as a DSB marker. Indeed, DNA-PK inhibition completely prevented resolution of 53BP1 foci up to 24 h post-irradiation in both 92.1 and OMM2.3 spheroid models compared to DMSO-proton irradiated spheroids (**Figures 5E, F**; **Supplementary Figures 13C, D**). ATM and ATR inhibition had a less pronounced effect but caused significant persistence of 53BP1 foci at 24–48 h post-irradiation in comparison to DMSO-irradiated spheroids. Collectively, this supports evidence that the radiosensitivity of UM spheroids to PBT in the presence of ATM, ATR and DNA-PK, similar to X-rays, is caused by persistence of DSB damage.

## 4 Discussion

PBT is a key, precision targeted treatment for UM, which can spare more of the normal healthy tissue surrounding the tumour when compared to conventional X-ray irradiation, because of the Bragg peak phenomenon. Despite this, challenges remain with acute and long-term adverse side effects experienced by patients. Utilising tumour radiosensitisation strategies would enable a lower radiation dose to be targeted to larger tumours, reducing potential radiation-induced visual loss and increasing eye retention rates. In this study, we have examined potent inhibitors of ATM, DNA-PK and ATR that coordinate and orchestrate the repair of DSB repair pathways that have been suggested as targets to sensitise and enhance UM cell death to radiotherapy (12). Using 3D spheroid models of UM, which more accurately replicate the tumour structure and how this impacts treatment in patients, we show that all three inhibitors, and particularly those targeting ATM and DNA-PK, can sensitise seven different UM models to X-rays and PBT, the magnitude of which is dependent on their inherent radiosensitivity. We also provide evidence that these radiosensitisation effects, as expected, were driven through the persistence of DSBs and chromosomal damage.

It has been previously shown that ATM inhibition (utilising an older generation inhibitor, KU-55933) sensitised four UM cell lines to both X-rays and PBT (6), correlating with our current findings shown here when utilising the more potent ATM inhibitor AZD1390. In fact, in the current study, we have extended these observations to using seven UM cell lines, but more importantly, in growing these as spheroid models in 3D where drug uptake and responses to radiation treatment are more accurate of patient responses as compared to 2D monolayers. Whilst other data in UM are scarce, the same inhibitor (AZD1390) used in this study has been shown to sensitise several other types of cancer in response to ionising radiation through suppression of DSB repair initiation (17, 26–28). Regarding DNA-PK, the inhibitor NU7441 has been previously been shown to significantly reduce the viability of OMM2.3 cells to γ-radiation, and also to cells that gain acquired resistance to the MEK-inhibitor, selumetinib (23). This supports our study in which we found that DNA-PK inhibition through AZD7648 appeared to yield the most potent radiosensitisation effect in combination with both X-rays and PBT. In fact, AZD7648 has been shown to be a potent radiosensitisation agent in various different tumour models through both *in vitro* and *in vivo* studies (17, 29–32). ATR inhibition appeared to be the weakest radiosensitiser of all three inhibitors used, although it was noticeable that this inhibitor also caused a significant decrease in relative growth and viability as a monotherapy. Hence, the concentration used for examining the synergy with radiation had to represent the most effective compromise between achieving measurable inhibition and avoiding unacceptable toxicity.

It was noticeable that in some cell lines, the DER with the inhibitors combined with PBT was lower than that compared to X-rays. For example, in Mel202 and MP41, the DERs for X-rays ranged from 3.2-9.5 and 1.4-10.0 whereas for PBT these were 1.5-5.0 and 1.6-5.2, respectively. This appeared to be because of an enhanced effect of PBT on spheroid growth compared to X-rays, likely due to the increased ionisation density (linear energy transfer) of the proton beam. Indeed, we recently reportedly using 28 MeV protons at the entrance dose results in a moderate increase in linear energy transfer of 2.6 keV/µm compared to X-rays which are ~1 keV/µm (24). However, there are also slight variations in the experimental setup, particularly with the horizontal beamline delivering protons compared to the vertical X-ray source, which need to be considered.

In terms of the mechanisms of action of the inhibitors post-irradiation, we confirmed that inhibiting the key kinases involved in the signalling and repair of DNA DSBs led to the persistence of the damage in UM spheroid models. Given that DNA-PK is the major protein co-ordinating the repair of the majority of DSBs throughout the cell cycle via NHEJ, it is not surprising that the inhibitor AZD7648 caused the most pronounced persistence of γH2AX and 53BP1 foci up to 24–48 h following both X-rays and PBT, and which aligned with DSB detection using neutral comet assays. Both ATM and ATR inhibition also, to a lesser extent than DNA-PK inhibition, caused a significant delay in the repair of DSBs as visualised by persistent γH2AX/53BP1 foci and persistent DNA DSBs in neutral comet assays. The persistence in DNA DSB damage with all three inhibitor-radiation combinations was accompanied by increases in the frequency of chromosomal aberrations, including micronuclei, nuclear budding, and nucleoplasmic bridges, highlighting the enhanced genome instability induced, and in the case of ATM and DNA-PK inhibition there was also a marked G2/M cell cycle arrest. No cell cycle arrest was detected following ATR inhibition post-irradiation, consistent with ATR’s known role in coordinating the G2/M checkpoint in response to DNA damage (33, 34), and demonstrates that chromosomal aberrations under these conditions were being caused by cells that were progressing through the cell cycle with unresolved DNA damage and impaired replication associated repair (35). Similar observations of persistent DSB damage and elevated micronucleus formation have been observed with AZD6738 in combination with both X-rays and PBT in other tumour models (17, 34).

In summary, data from our current study clearly show that inhibition of ATM, DNA-PK or ATR robustly radiosensitises UM 3D models to both X-ray irradiation and PBT by preventing the efficient repair of DNA DSBs, and which should be investigated further. In particular, there is a need to further understand any potential toxicity to normal intraocular cells (particularly the retina), to negate these where possible with targeted PBT. Future work could include the utilisation of more complex UM models, such as patient-derived organoids and experiments using mouse models *in vivo*, before introducing them into clinical care.

## Data Availability

The raw data supporting the conclusions of this article will be made available by the authors, without undue reservation.
