# Supplementary material for: Inhibition of ATM, ATR and DNA-PK radiosensitises 3D uveal melanoma models to X-rays and proton beam therapy
Source: Front Oncol. 2026 Aug 14;16:1921423. doi: 10.3389/fonc.2026.1921423 (PMC13521882; doi:10.3389/fonc.2026.1921423)
Supplement: Supplementary file 1 [file DataSheet1.docx]

Supplementary Material

**Supplementary Figure 1. ATM, DNA-PK and ATR inhibition suppresses UM spheroid growth in combination with X-rays.** (**A, D, G**) 92.1, (**B, E, H**) OMM2.3 or (**C, F, I**) Mel202 cells were seeded at 500 cells per well in an ultra-low attachment plate and spheroids left to form for 48 h. Following treatment with (**A-C**) ATMi (0.2 µM), (**D-F**) DNA-PKi (1 µM), (**G-I**) ATRi (1 µM for 92.1 and Mel202 spheroids, 2 μM for OMM2.3) or DMSO as a control for 1 h, cells were irradiated with 1 Gy X-rays. Data is expressed as the mean area (µm^3^)±SEM from three independent biological experiments containing triplicate spheroids.

**Supplementary Figure 2. ATM, DNA-PK and ATR inhibition suppresses UM spheroid growth and viability in combination with escalating X-ray doses**. (**A-C**) Mel270, (**D-F**) OMM1 or (**G-I**) MP46 cells were seeded at 500 cells per well in an ultra-low attachment plate and spheroids left to form for 48 h. UM spheroids were treated with ATMi (0.2 μM), DNA-PKi (1 µM), or ATRi (1 μM) for 1 h prior to X-ray irradiation (1, 2 or 4 Gy). (**A, D, G**) Relative changes in spheroid growth were assessed by measuring the change in area from day 3 to day 15 post-seeding, and normalised against the unirradiated control which was set to 1. (**B, E, H**) Cell viability was evaluated 7 days post-irradiation. (**C, F, I**) Representative images of spheroids on day 15 post-seeding. Data was acquired from three biologically independent experiments. *p<0.02, **p<0.01 as analysed by a two-sample *t*-test.

**Supplementary Figure 3**. **Validation of hydrogen peroxide treatment as a positive control for cell viability assays across UM spheroid models.** Cell viability was assessed in seven UM spheroid models with Cell Titer Glo 3D following 30 min treatment with 10 mM hydrogen peroxide (+) or DMSO as the vehicle control (−). Cell viability was quantified and values normalised to the vehicle only treated spheroids, which was set to 100 %. Data are shown as mean±SD. Spheroids were measured in triplicate with data shown from three independent biological experiments.

**Supplementary Figure 4. DNA repair inhibitors effectively suppress the activity of ATM, DNA-PK and ATR kinases in UM spheroids**. 92.1 or OMM1 cells were seeded at 500 cells per well in an ultra-low attachment plate and spheroids left to form for 48 h. Following treatment with (**A**) ATMi (0.2 µM), (**B**) DNA-PKi (1 µM), (**C**) ATRi (1 µM) or DMSO as a control for 1 h, cells were unirradiated or irradiated with 4 Gy X-rays. Whole cell extracts were prepared and levels of phosphorylation of (**A**) ATM at S1981, (**B**) DNA-PKcs at S2056 or (**C**) ATR at T1518 relative to the unmodified protein were analysed by immunoblotting. Relative quantification was performed by calculating the ratio of phosphorylated to total unphosphorylated protein, and normalising against the DMSO control which was set to 1.0.

**Supplementary Figure 5. Effect of ATM, DNA-PK and ATR inhibition alone on growth and viability of UM spheroid models**. (**A-B**) 92.1, (**C-D**) MP46, (**E-F**) Mel202, (**G-H**) OMM1, (**I-J**) Mel270, (**K-L**) OMM2.3 or (**M-N**) MP41 cells were seeded at 500 cells per well in an ultra-low attachment plate and spheroids left to form for 48 h, and then treated with ATMi (0.2 µM), DNA-PKi (1 µM), ATRi (1 µM) or DMSO as a control for 1 h. (**A, C, E, G, I, K, M**) Relative changes in spheroid growth were assessed by measuring the change in area from day 3 to day 15 post-seeding, and normalised against the unirradiated control which was set to 1.0. (**B, D, F, H, J, L, N**) Cell viability was evaluated 7 days post-treatment and normalised against the DMSO control which was set to 1.0. Data was acquired from three biologically independent experiments. *p<0.02, **p<0.01, ***p<0.005 as analysed by a two-sample *t*-test.

**Supplementary Figure 6. Inhibition of ATM, DNA-PK and ATR causes delayed and persistent DSBs after X-ray irradiation.** (**A**) 92.1 and (**B**) OMM2.3 cells were seeded at 500 cells per well in an ultra-low attachment plate and spheroids left to form for 48 h, and then treated with ATMi (0.2 µM), DNA-PKi (1 µM) or ATRi (1 µM for 92.1 and 2 µM for OMM2.3) or DMSO as a control for 1 h. Cells were irradiated with 4 Gy of X-rays, DNA DSBs measured using the neutral comet assay. Shown are the representative images for unirradiated controls, and 0 and 4 h post radiation.

**Supplementary Figure 7. Inhibition of ATM, DNA-PK or ATR causes delayed and persistent DSBs after X-ray irradiation.** (**A** and **C**) 92.1 and (**B** and **D**) OMM2.3 cells were seeded at 500 cells per well in an ultra-low attachment plate and spheroids left to form for 48 h, and then treated with ATMi (0.2 µM), DNA-PKi (1 µM), ATRi (1 µM for 92.1 and 2 µM for OMM2.3) or DMSO as a control for 1 h. DNA DSBs were measured by immunofluorescence microscopy using γH2AX and 53BP1 foci as surrogate markers at (**A-B**) 1 h and (**C-D**) 24 h post-irradiation, where representative images are shown. Scale bar is 12 μm.

**Supplementary Figure 8. Inhibition of ATM, DNA-PK and ATR causes delayed and persistent DSBs processed by HR after X-ray irradiation.** (**A** and **C**) 92.1 and (**B** and **D**) OMM2.3 cells were seeded at 500 cells per well in an ultra-low attachment plate and spheroids left to form for 48 h, and then treated with ATMi (0.2 µM), DNA-PKi (1 µM), ATRi (1 µM for 92.1 and 2 µM for OMM2.3) or DMSO as a control for 1 h. DNA DSBs processed by HR were measured by immunofluorescence microscopy through Rad51 foci in mitosin positive cells at (**A-B**) 8 h and (**C-D**) 24 h post-irradiation, where representative images are shown. Scale bar is 12 μm.

**Supplementary Figure 9.** **Cell cycle analysis of UM spheroids following ATM, DNA-PK and ATR inhibition post X-ray irradiation.** (**A-D)** 92.1 and (**E-F**) OMM2.3 cells were seeded at 500 cells per well in an ultra-low attachment plate and spheroids left to form for 48 h, and then treated with (**B, F**) ATMi (0.2 µM), (**C, G**) DNA-PKi (1 µM), (**D, H**) ATRi (1 µM for 92.1 and 2 µM for OMM2.3) or (**A, E**) DMSO as a control for 1 h. Cells were irradiated with 4 Gy of X-rays, left for 24 h to repair, and the cell cycle analysis performed by flow cytoemetry. Cell cycle phase were gated consistently across all samples using Floreida.io. Quantification was performed using a minimum of 5,000 cells per sample.

**Supplementary Figure 10. ATM, DNA-PK and ATR inhibition suppresses UM spheroid growth in combination with PBT.** (**A, D, G**) 92.1, (**B, E, H**) OMM2.3 or (**C, F, I**) Mel202 cells were seeded at 500 cells per well in an ultra-low attachment plate and spheroids left to form for 48 h. Following treatment with (**A-C**) ATMi (0.2 µM), (**D-F**) DNA-PKi (1 µM), or (**G-I**) ATRi (1 µM for 92.1 and Mel202 spheroids or 2 μM for OMM2.3) or DMSO as a control for 1 h, cells were irradiated with 1 Gy PBT. Data is expressed as the mean area (µm^3^)±SEM from three independent biological experiments containing triplicate spheroids.

**Supplementary Figure 11. ATM, DNA-PK or ATR inhibition suppresses UM spheroid growth and viability in combination with escalating PBT doses**. (**A-C**) Mel270, (**D-F**) MP46 or (**G-I**) OMM1 cells were seeded at 500 cells per well in an ultra-low attachment plate and spheroids left to form for 48 h. UM spheroids were treated with ATMi (0.2 μM), DNA-PK (1 µM), or ATRi (1 μM) for 1 h prior to PBT (1, 2 or 3 Gy). (**A, D, G**) Relative changes in spheroid growth were assessed by measuring the change in area from day 3 to day 15 post-seeding, and normalising against the unirradiated control which was set to 1. (**B, E, H**) Cell viability was evaluated 7 days post-irradiation. (**C, F, I**) Representative images of spheroids on day 15 post-seeding. Data was acquired from three biologically independent experiments. *p<0.02, **p<0.01 as analysed by a two-sample *t*-test.

**Supplementary Figure 12. Inhibition of ATM, DNA-PK and ATR causes delayed and persistent DSBs after PBT.** (**A**) 92.1 and (**B**) OMM2.3 cells were seeded at 500 cells per well in an ultra-low attachment plate and spheroids left to form for 48 h, and then treated with ATMi (0.2 µM), DNA-PKi (1 µM) or ATRi (1 µM for 92.1 and 2 µM for OMM2.3) or DMSO as a control for 1 h. Cells were irradiated with 4 Gy of PBT, DNA DSBs measured using the neutral comet assay. Shown are the representative images for unirradiated controls, and 0 and 4 h post radiation.

**Supplementary Figure 13.** **Inhibition of ATM, DNA-PK and ATR causes delayed and persistent DSBs after PBT.** (**A** and **C**) 92.1 and (**B** and **D**) OMM2.3 cells were seeded at 500 cells per well in an ultra-low attachment plate and spheroids left to form for 48 h, and then treated with ATMi (0.2 µM), DNA-PKi (1 µM), ATRi (1 µM for 92.1 and 2 µM for OMM2.3) or DMSO as a control for 1 h. DNA DSBs were measured by immunofluorescence microscopy using γH2AX and 53BP1 foci as surrogate markers at (**A-B**) 1 h and (**C-D**) 24 h post-irradiation, where representative images are shown. Scale bar is 12 μm.
